# Does the CRP/Albumin Ratio Independently Predict Survival in Metastatic Colorectal Cancer? Insights from a Propensity Score-Matched Cohort

**DOI:** 10.3390/cancers18152425

**Published:** 2026-07-28

**Authors:** Mehmet Cem Fidan, Murad Guliyev, Emir Çerme, Hamza Abbasov, Vali Aliyev, Berrin Papila, Nebi Serkan Demirci, Özkan Alan, Çiğdem Papila

**Affiliations:** 1 Division of Medical Oncology, Department of Internal Medicine, Cerrahpaşa Faculty of Medicine, Istanbul University-Cerrahpaşa, Istanbul 34098, Türkiye; drmuradguliyev@gmail.com (M.G.); emircermemd@gmail.com (E.Ç.); hamza.abbasov@iuc.edu.tr (H.A.); dktraliyev@gmail.com (V.A.); nebiserkan.demirci@iuc.edu.tr (N.S.D.); ozkan.alan@iuc.edu.tr (Ö.A.); bpapila@iuc.edu.tr (Ç.P.); 2 Department of General Surgery, Cerrahpaşa Faculty of Medicine, Istanbul University-Cerrahpaşa, Istanbul 34098, Türkiye; berrin.papila@iuc.edu.tr

**Keywords:** CRP/albumin ratio, colorectal cancer, propensity score matching, prognosis, inflammation

## Abstract

**Background:** The C-reactive protein/albumin ratio (CAR) has emerged as a prognostic biomarker across several malignancies, but its role in de novo metastatic colorectal cancer (mCRC) has not been evaluated using a propensity score-matched design. **Methods:** This retrospective study included 212 patients with mCRC who received first-line fluoropyrimidine-based chemotherapy plus a biological agent. The optimal CAR cutoff (2.69) was determined by ROC analysis (AUC = 0.714). Propensity score matching (PSM), incorporating 16 clinical, pathological, and molecular covariates, was performed to balance low-CAR and high-CAR groups. Progression-free survival (PFS) and overall survival (OS) were compared using Kaplan–Meier and multivariate Cox regression analyses. **Results:** PSM yielded 65 matched pairs (130 patients), eliminating all baseline imbalances present in the unmatched cohort. In the matched cohort, high CAR remained associated with shorter PFS (median 8.4 vs. 12.9 months; HR = 1.58, 95% CI 1.10–2.30, *p* = 0.014) and OS (median 22.7 vs. 31.1 months; HR = 1.70, 95% CI 1.15–2.50, *p* = 0.006). On multivariate analysis, CAR retained independent significance for OS (HR = 1.792, 95% CI 1.157–2.775, *p* = 0.009) alongside CEA level (HR = 3.331, *p* < 0.001) and BRAF mutation status (HR = 4.543, *p* = 0.040), but not for PFS, where mucinous component (HR = 2.335, *p* = 0.015) and CEA level (HR = 2.231, *p* = 0.045) were the dominant independent predictors. **Conclusions:** Baseline CAR is an independent prognostic factor for OS in mCRC, even after adjusting for established clinicopathological and molecular risk factors through propensity score matching. As an inexpensive and readily available biomarker, CAR may help identify patients with more aggressive disease biology who warrant closer monitoring, although prospective validation is needed before clinical implementation.

## Simple Summary

Colorectal cancer is one of the most common and deadly cancers worldwide, and about one in five patients already has the disease that has spread to other organs at diagnosis. For these patients, clinicians need simple, inexpensive tools to estimate how aggressive the disease is likely to be. The C-reactive protein-to-albumin ratio is calculated from two routine blood tests that together reflect a patient’s inflammation and nutritional state. In this study of 212 patients with newly diagnosed metastatic colorectal cancer, we examined whether a high ratio before treatment predicts survival. Using a statistical matching method to balance many clinical and tumor characteristics between groups, we found that a high ratio was independently associated with shorter overall survival, even after accounting for established risk factors. This cheap, readily available marker may help identify patients with more aggressive disease who need closer monitoring, although further studies are needed before routine use.

## 1. Introduction

Colorectal cancer (CRC) is the second leading cause of cancer-related deaths worldwide, causing approximately one million deaths annually [1]. Distant metastases are present at the time of initial diagnosis in approximately 21% of patients, and the five-year relative survival rate for these patients is less than 15% [2]. For decades, treatment options for metastatic CRC have been primarily limited to systemic chemotherapy. However, the inclusion of targeted therapies against the epidermal growth factor receptor (EGFR) and vascular endothelial growth factor receptor (VEGFR) signaling pathways, as well as immune checkpoint inhibitors (ICIs), has led to improved clinical outcomes in selected patient populations [3,4,5,6]. Current standard care treatments have significantly improved mean overall survival in prospective clinical trials: In RAS-wild-type tumors, the duration reached approximately 40 months; in RAS-mutated tumors, 25–28 months; in patients with deficient mismatch repair (dMMR) or high microsatellite instability tumors, more than 75 months; and in the subgroup with poor prognosis BRAFV600E mutations, approximately 18 months [7,8,9,10]. In the diagnostic phase, which forms one of the foundations of the oncological process, tumor characterization can be understood through comprehensive molecular analyses of biopsy samples, including microsatellite instability and RAS/BRAF mutation status. This is crucial for guiding treatment choices in metastatic colorectal cancer.

Inflammation is a significant component of cancer development and progression [11,12]. The prognostic significance of peripheral blood-based inflammatory biomarkers is attracting attention because of their ease of use, cost-effectiveness, and potential for dynamic monitoring, given the relationship between inflammation and cancer pathogenesis and progression. The C-reactive protein (CRP)/albumin ratio (CAR), a composite biomarker reflecting systemic inflammation (via CRP) and nutritional status (via albumin), has emerged as a strong prognostic indicator in various malignancies, including CRC [13,14,15,16,17]. Previous studies have shown that a high CAR may be associated with poor survival outcomes in patients with metastatic CRC [18,19,20].

To our knowledge, no prospective cohort study has been conducted, given that previous studies evaluating the prognostic significance of CAR in metastatic CRC may be affected by selection bias and confounding variables due to their retrospective designs. Therefore, it is essential to investigate the prognostic value of this biomarker using a more robust analysis. Propensity score-based analyses are increasingly used to estimate the effects of various treatments in observational studies [21]. Propensity score methods reduce confounding effects from measured baseline variables by creating a paired or weighted sample in which the distribution of measured baseline variables is similar across study groups. This allows for an accurate assessment of outcomes across groups and the use of treatment effect measures, such as those used in randomized controlled trials [22].

In this study, we aimed to evaluate the prognostic significance of CAR in a clinically more homogeneous subgroup consisting only of newly diagnosed metastatic colorectal cancer patients receiving first-line fluoropyrimidine-based chemotherapy in combination with a biological agent. Additionally, we used propensity score matching to minimize baseline disparities and robustly assess the association between CAR and survival outcomes in this patient population.

## 2. Materials and Methods

### 2.1. Study Design and Patients

This retrospective study included 212 patients with metastatic colorectal cancer who were diagnosed and followed up at the Department of Medical Oncology, Cerrahpaşa Faculty of Medicine, Istanbul University-Cerrahpaşa, between January 2013 and May 2025. The patients included had a histopathologically confirmed diagnosis of colorectal adenocarcinoma, radiologically confirmed distant metastasis at the time of diagnosis, received first-line fluoropyrimidine-based chemotherapy plus a biological agent (bevacizumab, cetuximab, or panitumumab), and had pre-treatment peripheral blood sample results available. Surgery targeting the primary tumor refers to surgery performed after systemic treatment. Patients with concomitant malignancy, non-adenocarcinoma histology (squamous cell carcinoma, neuroendocrine carcinoma, etc.), pre-treatment active infection, liver failure, and those with unavailable inflammatory laboratory tests or insufficient clinical/survival data were excluded from the study.

Demographic, clinicopathological, treatment, and laboratory data were collected from clinical records, and treatment regimens and dosages were consistent with baseline clinical trial protocols [4,5,6]. CAR was calculated as the ratio of serum CRP (mg/L) to albumin (g/dL). CRP and albumin were measured as part of routine clinical care in the hospital’s accredited central biochemistry laboratory. Owing to the retrospective design spanning [2013–2025], the specific analyzers and reagents may have varied over the study period and could not be uniformly retrieved; all measurements were performed using standard, quality-controlled clinical assays. Patients were grouped as low CAR (≤2.69; *n* = 88) and high CAR (>2.69; *n* = 124) according to the cutoff value determined by ROC analysis, which evaluated the discriminative power in predicting death (CAR = 2.69; AUC = 0.714, 95% CI 0.623–0.804; *p* = 0.001). This study was designed, conducted, and reported in accordance with the STROBE (Strengthening the Reporting of Observational Studies in Epidemiology) guidelines for observational cohort studies [23].

### 2.2. Efficacy and Safety Measures

The time from the first treatment administration to disease progression or death from any cause, whichever occurred first, was defined as progression-free survival (PFS). Disease progression was determined based on radiological reports and clinical assessments recorded in the medical records. Patients who did not experience disease progression were censored at the last follow-up visit. Overall survival (OS) was defined as the time from the first treatment administration to death from any cause or the last visit date. We censored patients who were still alive on the date of analysis.

### 2.3. Ethical Considerations

This research was conducted in accordance with the principles outlined in the Helsinki Declaration and received approval from the local clinical research ethics committee (date: December 2024; number: E-83045809-604.01-1187399). Given the retrospective nature of this study, the requirement for informed consent was waived.

### 2.4. Statistical Analysis

Categorical variables are presented as frequencies and percentages. The optimal CAR cutoff was determined as the value maximizing the Youden index on the ROC curve for overall survival, and patients were dichotomized accordingly. Missing data (3.3–41.5%) were completed using the Multiple Imputation module of IBM SPSS Statistics (version 27.0.1.0) with full conditional specification (FCS) using chained equations (5 imputation datasets) [24]. The highest proportion of missing values was observed for tumor differentiation (41.5%), followed by smoking status (32.1%) and mucinous component (24.5%), while RAS status had the lowest (3.3%). Propensity score matching (PSM) was used to reduce confounding effects between the CAR groups. The propensity score was calculated using multivariate logistic regression with 16 clinical, pathological, and molecular covariates and averaged over five imputations. Patients were matched 1:1 without substitution using the greedy nearest-neighbor algorithm on logit (PS) with the SPSS PS Matching extension (SPSSINC PS MATCH), and caliper = 0.19 (0.2 times logit PS standard deviation [SD]) [25]. Equilibrium was assessed using the standardized mean difference (SMD < 0.10) [25]; 65 pairs (130 patients) were matched for analysis.

Survival analyses were performed using the Kaplan–Meier and log-rank tests. The effect of CAR on PFS and OS was evaluated in the matched cohort (n = 130) using multivariate Cox regression. Variables were selected for the multivariate Cox models based on clinical relevance and univariable significance (*p* < 0.20); these candidate variables, together with the four covariates that remained imbalanced after matching, were entered into the final models. Because propensity scores were averaged over the five imputations to generate a single matched cohort, the imbalanced covariates (BRAF, sex, tumor grade, mucinous histology) were entered as their MICE-imputed values within this matched cohort rather than as case-wise complete data, avoiding listwise deletion [24]. Hazard ratios were estimated in this single matched cohort and reported with 95% confidence intervals; separate per-imputation pooling by Rubin’s rules was therefore not required. All analyses were performed using SPSS 27.0.1.0; *p* < 0.05 was considered significant.

The complete (*n* = 212) cohort dataset, with variable descriptions, is presented in Table 1.

## 3. Results

### 3.1. Characteristics of Patients

A total of 212 patients with metastatic colorectal cancer were included in this study. The median age of the patients was 61 years (range: 21–85). There were 124 (58.5%) male and 88 (41.2%) female patients in the study. The distribution of primary tumor locations was as follows: 61% in the left colon, 24% in the right colon, and 15% in the rectum. All patients were diagnosed with adenocarcinoma according to histopathological evaluation, and distant metastases were confirmed radiologically at the time of the initial diagnosis. The most frequent site of metastasis was the liver (73.6%), followed by the peritoneum (26.4%) and lungs (20.8%) (Table 1).

Of these patients, 88 (41.5%) were in the low-CAR group and 124 (58.5%) were in the high-CAR group. In the pre-matching comparison, the two groups were similar in terms of age, sex, smoking status, BMI, presence of mucinous component, tumor differentiation, primary tumor localization, RAS/BRAF/MSI mutation status, and applied treatment regimen (all *p* > 0.05). However, significant differences were found in six variables: the high-CAR group had a higher proportion of patients with ECOG performance status ≥1, a higher proportion of patients with CEA and CA19-9 levels above the upper limit of normal, a higher frequency of liver metastasis, and involvement of more than two metastatic sites, while the proportion of those who underwent primary tumor resection was lower (Table 1).

**Figure 1:**
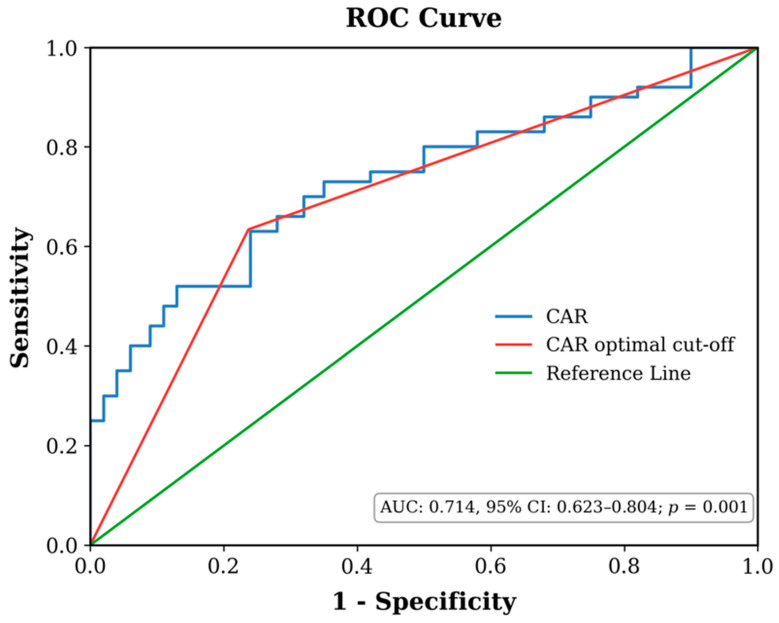
ROC curve for CAR in the prediction of overall survival. CAR: C-reactive protein to albumin ratio; AUC: area under the curve; ROC: receiver operating characteristic; CI: confidence interval.

According to ROC analysis, when OS was used as the endpoint, the optimal cutoff value for CAR was 2.69 (AUC: 0.714, 95% CI: 0.623–0.804, *p* = 0.001) (Figure 1). Patients were divided into two groups: low CAR (<2.69) and high CAR (>2.69).

To address these baseline imbalances, propensity score matching resulted in 65 pairs and 130 patients. In the matched cohort (Table 2), there were no statistically significant differences between the two groups for any baseline characteristics, including all variables that previously showed significant differences (all *p* > 0.05).

### 3.2. Treatment Interventions

All patients included in the study received fluoropyrimidine-based chemotherapy in combination with a biological agent as the first-line treatment. Among the fluoropyrimidine derivatives, 5-fluorouracil was administered to 165 patients (77.8%), and capecitabine was administered to 47 patients (22.2%). Oxaliplatin was administered in combination with fluoropyrimidine to 144 patients (67.9%), and irinotecan to 68 patients (32.1%). No patient received FOLFOXIRI as the first-line treatment regimen. The most frequently administered biological agent was bevacizumab (n = 155; 73.1%), followed by cetuximab (*n* = 43; 20.3%) and panitumumab (*n* = 14; 6.6%). The patients received an average of two treatment series (range: 1–5). Detailed information on the subsequent treatment regimens is provided in Supplementary Table S1.

### 3.3. Survival Analyses

**Figure 2:**
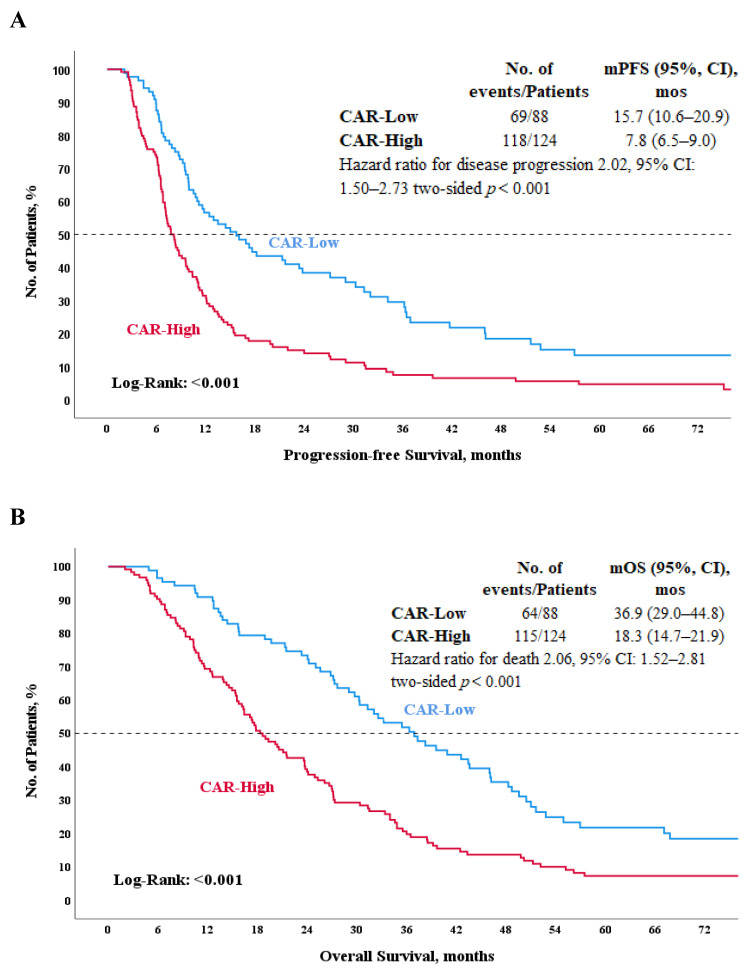
Kaplan–Meier curves for (**A**) progression-free survival and (**B**) overall survival according to CAR group in the unmatched cohort (*n* = 212). CAR: C-reactive protein/albumin ratio. Dashed lines indicate median survival.

In the entire pre-matched cohort (*n* = 212), the median follow-up time was 24.5 months (min-max: 3.1–138.8 months). In this study, 88.2% of patients experienced disease progression after first-line treatment. Both PFS (median 7.8 vs. 15.7 months; HR = 2.02; 95% CI 1.50–2.73; *p* < 0.001) and OS (median 18.3 vs. 36.9 months; HR = 2.06; 95% CI 1.52–2.81; *p* < 0.001) were significantly shorter in the high-CAR group than in the low-CAR group (Figure 2).

**Figure 3:**
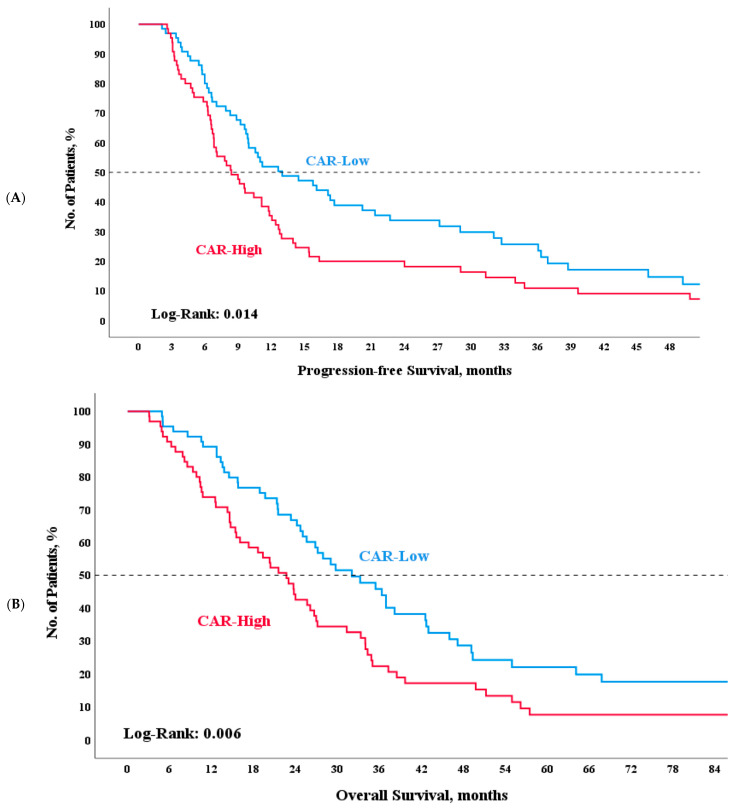
Kaplan–Meier curves for (**A**) progression-free survival and (**B**) overall survival according to CAR group in the propensity score-matched cohort (*n* = 130). CAR: C-reactive protein/albumin ratio. Dashed lines indicate median survival.

In the propensity score-matched cohort (*n* = 130), both PFS (median 8.4 vs. 12.9 months; HR = 1.58; 95% CI 1.10–2.30; *p* = 0.014) and OS (median 22.7 vs. 31.1 months; HR = 1.70; 95% CI 1.15–2.50; *p* = 0.006) were significantly shorter in the high-CAR group than in the low-CAR group (Figure 3).

### 3.4. Multivariate Cox Regression Analyses

In the multivariate Cox regression analysis performed on the matched cohort, the presence of a mucinous component (HR = 2.335; 95% CI 1.178–4.628; *p* = 0.015) and CEA levels above the upper limit (HR = 2.231; 95% CI 1.017–4.895; *p* = 0.045) were identified as independent prognostic factors for PFS. Although CAR status was significant in the univariate analysis, it did not retain its significance in the multivariate analysis (Table 3).

In the Cox regression analyses for OS, CAR status (HR = 1.792; 95% CI 1.157–2.775; *p* = 0.009), CEA level (HR = 3.331; 95% CI 1.830–6.065; *p* < 0.001), and BRAF mutation status (HR = 4.543; 95% CI 1.070–19.287; *p* = 0.040) were identified as independent prognostic factors (Table 4). In a sensitivity analysis treating CAR as a continuous variable, higher CAR was significantly associated with worse OS (HR = 1.030 per unit, 95% CI 1.018–1.042, *p* < 0.001).

## 4. Discussion

This retrospective study, which included 212 patients with mCRC and used propensity score matching, demonstrated that a high baseline CAR value (>2.69) was associated with shorter PFS and OS with a pre-matched cutoff. Before matching, the high CAR group was characterized by a higher incidence of ECOG performance status ≥1, liver metastasis, ≥2 metastatic site involvement, elevated CEA/CA 19-9, and a lower primary tumor resection rate, suggesting that high CAR may reflect not only an inflammatory state but also a more advanced and symptomatic disease stage. To our knowledge, this is the first study to evaluate the prognostic role of CAR, specifically in a mCRC population, using a propensity score-matched design.

The biological potential of CAR as a prognostic tool is based on its involvement in well-known inflammatory pathways. Inflammation facilitates malignant progression by promoting angiogenesis and lymphangiogenesis in the tumor microenvironment [26]. CRP, an acute-phase reactant induced by IL-1, IL-6, and TNF-α, increases in parallel with inflammatory activity [27], whereas the same cytokine environment simultaneously suppresses albumin synthesis [28]. Therefore, CAR functions as a composite indicator of two interconnected but opposing processes, which may explain CAR’s consistently low-cost performance as a prognostic marker in different tumor types [29,30]. Our findings are largely consistent with the results of a meta-analysis by Liao et al., which included 6329 patients with CRC across all stages and showed that a high pre-treatment CAR was associated with worse OS and PFS [15].

A significant methodological difference in this study is the use of PSM, which includes patient characteristics such as age, sex, ECOG performance status, and BMI; tumor characteristics such as tumor differentiation, mucinous component status, BRAF, RAS, and MSI; disease characteristics such as pretreatment CEA and CA 19-9 levels, presence of liver or peritoneal metastasis, number of metastatic sites, and primary tumor resection status; and the regimen administered as covariates to balance CAR groups before survival comparison. This adjustment successfully balanced all variables that previously showed significant differences between groups, including ECOG performance status, primary tumor resection, liver metastasis burden, CEA, and CA 19-9; after matching, no statistically significant differences remained in any of these variables in the cohort. This finding demonstrates that the PS model provides an effective balance in terms of the selected covariates and that subsequent survival comparisons can be interpreted independently of these measurable covariates. Because tumor location was included as a matching covariate, both colon and rectal primaries were balanced across CAR groups after propensity score matching, supporting the combined analysis of these anatomically related entities that share first-line systemic treatment principles in the de novo metastatic setting.

In the current literature, data on CAR in patients with metastatic colorectal cancer generally consist of retrospective studies with small sample sizes. One study showed that a high pre-treatment CAR was associated with worse OS in patients with unresectable metastatic colorectal cancer, and in this study, a trend toward improved survival was observed in patients whose post-treatment CAR normalized compared to those whose CAR remained high both before and after treatment [18]. A large pooled analysis encompassing six studies and 771 patients confirmed that a high CAR was associated with worse OS and PFS [20]. It should be noted that CAR values depend on the unit convention used for CRP measurement (mg/L vs. mg/L); accordingly, the numerical cutoff identified here (with CRP expressed in mg/L) is not directly comparable with studies expressing CRP in mg/L. Reported CAR cut-offs in colorectal cancer vary widely (approximately 0.03–0.67 in a large meta-analysis [15]), largely reflecting differences in CRP unit conventions and assay methods; notably, that meta-analysis found the prognostic value of CAR to be preserved irrespective of the specific cut-off applied. This variability underscores the need for a standardized, prospectively validated threshold before clinical use.

Our survival predictions in the matched cohort are consistent with the literature: a high CAR remained associated with shorter PFS and OS even after matching. In the multivariate analysis, CAR retained its independent significance for OS but not for PFS. At this point, the presence of a mucinous component and high CEA levels became the dominant independent predictors of PFS. The consistent association between high CAR and CEA levels in our study, as shown in previous studies [15], suggests that part of the effect of CAR on PFS may be related to this association. The independent negative effect of the mucinous component on PFS can be explained by the association between this histological subtype and chemotherapy resistance [31]. The divergence between CAR’s effect on overall survival (OS) and its lack of an independent effect on PFS under first-line treatment may be based on a biological foundation. PFS is largely governed by the intrinsic biology of the tumor and its sensitivity to chemotherapy; this was better captured in our cohort by mucosal histology and CEA levels. In contrast, a high CAR reflects systemic inflammation and impaired nutritional status that accumulate throughout the disease course and are closely associated with cachexia, sarcopenia, and decreased performance status. This catabolic state can compromise the patient’s ability to tolerate and access lines of treatment beyond first-line progression, thus having a stronger impact on overall mortality than the timing of the initial radiological progression.

In the multivariate analysis for OS, CAR, BRAF mutation status, and high pre-treatment CEA levels were identified as independent prognostic factors. The association between BRAF mutations and poor prognosis in metastatic CRC has been established previously [32]. Although the direction of this association is consistent with the literature, only three BRAF-mutant patients were present in the matched cohort; the multivariate model is therefore markedly underpowered for BRAF, the very wide confidence interval (1.070–19.287) should be interpreted with caution, and this estimate must be regarded as hypothesis-generating rather than confirmatory. Similarly, the independent negative effect of high CEA levels on OS was consistent with that of previous studies [33,34]. When these findings are considered together, the fact that CAR retains its significance independently of established, biologically robust prognostic factors such as BRAF and CEA in our multivariate model for OS suggests that CAR provides complementary prognostic information beyond these markers.

Previous studies linking CAR to outcomes in metastatic CRC have predominantly focused on patients who underwent curative resection for liver metastases [19]. Our cohort differed in that it encompassed a larger and possibly more representative population: 55.7% of patients never underwent surgical resection, while the remaining 44.3% did. In the unmatched cohort, primary tumor resection was performed significantly more frequently in the low-CAR group than in the high-CAR group (55.7% vs. 36.3%); this difference disappeared entirely after PSM. By encompassing both resectable and unresectable diseases rather than focusing solely on a surgically selected subgroup, our findings may be more easily generalizable to the general patient population of mCRC in daily clinical practice. In the surgically treated subgroup, the extent and quality of locoregional resection—including nodal assessment, which can be enhanced by intraoperative techniques such as indocyanine green fluorescence—may influence outcomes but were not captured in the present analysis [35].

Our study has several limitations despite the use of PSM. Its generalizability is limited because of its single-center, retrospective design. In our study, CAR was only recorded pre-treatment and not evaluated post-treatment or throughout the treatment process, preventing the assessment of whether dynamic changes in CAR carry prognostic information. Prospective studies, including repeated pre- and post-treatment CAR measurements, would help clarify this issue. Furthermore, there was a significant lack of data on BRAF and MSI mutation status, which is important for reflecting the full power of the model we established regarding the variables and limiting the impact of molecular subgroups on survival outcomes. The complication status of the primary tumor (e.g., obstruction, perforation, or bleeding) and the presence or absence of tumor-related symptoms were not systematically documented and thus could not be incorporated as covariates; these factors may influence disease behavior and warrant assessment in prospective studies.

## 5. Conclusions

The baseline CAR is an inexpensive and readily available biomarker that reflects the severity of the systemic inflammatory response in patients with mCRC. It should not be considered a treatment selection tool alone; however, a high CAR, as an additional factor to established clinicopathological risk factors (such as BRAF mutation status, CEA level, and mucinous histology), can help identify a subgroup of patients with more aggressive disease biology who may require closer clinical follow-up and more comprehensive prognostic evaluation. Prospective validation studies are needed before the CAR can be formally incorporated into clinical decision-making processes.

## Figures and Tables

**Table 1:** Baseline characteristics of patients by CAR groups before propensity score matching (n = 212).

Variables			Low-CAR	High-CAR	*p*	
		*n*	(*n* = 88)	(*n* = 124)		
Age, median (min.–max.)	61 (21–85)		60 (22–79)	62 (21–85)	0.164	m
Age (years), *n* (%)	<65	131 (61.8)	55 (62.5)	76 (61.3)	0.858	X^2^
	≥65	81 (38.2)	33 (37.5)	48 (38.7)		
Gender *n* (%)	Female	88 (41.5)	41 (46.6)	47 (37.9)	0.206	X^2^
	Male	124 (58.5)	47 (53.4)	77 (62.1)		
ECOG, *n* (%)	0	85 (40.1)	46 (52.3)	39 (31.5)	***0.004***	X^2^
	≥1	127 (59.9)	42 (47.7)	85 (68.5)		
Smoking status, *n* (%)	No	83 (39.2)	35 (39.8)	48 (38.7)	0.252	X^2^
	Yes	61 (28.8)	20 (22.7)	41 (33.1)		
	Unknown	68 (32.1)	33 (37.5)	25 (20.2)		
BMI, *n* (%)	≥25	109 (51.4)	37 (42.0)	62 (50.0)	0.799	X^2^
	<25	79 (37.3)	31 (35.2)	48 (38.7)		
	Unknown	34 (16.0)	20 (22.7)	14 (11.3)		
Mucinous component, *n* (%)	No	115 (54.2)	52 (59.1)	63 (50.8)	0.869	X^2^
	Yes	45 (21.2)	21 (23.9)	24 (19.4)		
	Missing	52 (24.5)	15 (17.0)	37 (29.8)		
Tumor differentiation, *n* (%)	Well	85 (40.1)	42 (47.7)	43 (34.7)	0.736	X^2^
	Moderate or poor	39 (18.4)	18 (20.5)	21 (16.9)		
	Unknown	88 (41.5)	28 (31.8)	60 (48.4)		
Location of primary tumor, *n* (%)	Left colon	130 (61.3)	58 (65.9)	72 (58.1)	0.486	X^2^
	Right colon	50 (23.6)	19 (21.6)	31 (25)		
	Rectum	32 (15.1)	11 (12.5)	21 (16.9)		
Primary tumor resection *, *n* (%)	No	118 (55.7)	39 (44.3)	79 (63.7)	***0.005***	X^2^
	Yes	94 (44.3)	49 (55.7)	45 (36.3)		
No. of metastatic sites	1	137 (64.6)	66 (75.0)	71 (57.3)	***0.008***	X^2^
	≥2	75 (35.4)	22 (25.0)	53 (42.7)		
RAS status, *n* (%)	Mutant	99 (46.7)	41 (46.6)	58 (46.8)	0.902	X^2^
	Wild	106 (50.0)	43 (48.9)	63 (50.8)		
	Unknown	7 (3.3)	4 (4.5)	3 (2.4)		
BRAF status, *n* (%)	Mutant	6 (2.8)	1 (1.1)	5 (4.0)	0.240	Fisher’s exact
	Wild	167 (78.8)	73 (83.0)	94 (75.8)		
	Unknown	39 (18.4)	14 (15.9)	25 (20.2)		
MSI status, *n* (%)	High	7 (3.3)	2 (2.3)	5 (4.0)	0.701	Fisher’s exact
	Stable	171 (80.7)	73 (83.0)	68 (54.8)		
	Unknown	34 (16.0)	13 (14.8)	21 (16.9)		
CEA level, *n* (%)	<ULN	44 (20.8)	29 (33.0)	15 (12.1)	***<0.001***	X^2^
	≥ULN	134 (63.2)	47 (53.4)	87 (70.2)		
	Unknown	34 (16.0)	12 (13.6)	22 (17.7)		
CA 19-9 level, *n* (%)	<ULN	76 (35.8)	43 (48.9)	33 (26.6)	***0.002***	X^2^
	≥ULN	102 (48.1)	34 (38.6)	68 (54.8)		
	Unknown	34 (16.0)	11 (12.5)	23 (18.5)		
Treatment regimens, *n* (%)	Oxaliplatin-based	144 (67.9)	65 (73.9)	79 (63.7)	0.119	X^2^
	Irinotecan-based	68 (32.1)	23 (26.1)	45 (36.3)		
Liver metastasis, *n* (%)	No	56 (26.4)	30 (34.1)	26 (21.0)	***0.033***	X^2^
	Yes	156 (73.6)	58 (65.9)	98 (79.0)		
Peritoneal metastasis, *n* (%)	No	156 (73.6)	64 (72.7)	92 (74.2)	0.811	X^2^
	Yes	56 (26.4)	24 (27.3)	32 (25.8)		

Bold-italic values indicate statistical significance (*p* < 0.05). BMI: body mass index; CEA: Carcinoembryonic antigen; CA 19-9: Carbohydrate antigen 19-9; CAR: C-reactive protein (CRP)/albumin ratio; ECOG: Eastern Cooperative Oncology Group; MSI: Microsatellite instability; m: Mann–Whitney u; *n*: Number; ULN: Upper limit of normal; Primary tumor resection refers to surgery performed after systemic therapy in patients with de novo metastatic disease; X^2^: Chi-square. * Charlson Comorbidity Index was defined by only comorbidities without adjusting age.

**Table 2:** Baseline characteristics of patients by CAR groups after propensity score matching (n = 130).

Variables			Low-CAR	High-CAR	*p*	
		*n*	(*n* = 65)	(*n* = 65)		
Age, median (min.–max.)	62 (21–85)		62 (22–79)	63 (21–85)	0.510	m
Age (years), *n* (%)	<65	74 (56.9)	37 (56.9)	37 (56.9)	1.000	X^2^
	≥65	56 (43.1)	28 (43.1)	28 (43.1)		
Gender *n* (%)	Female	56 (43.1)	30 (46.2)	26 (40.0)	0.479	X^2^
	Male	74 (56.9)	35 (53.8)	39 (60.0)		
ECOG, *n* (%)	0	56 (43.1)	27 (41.5)	29 (44.6)	0.723	X^2^
	≥1	74 (56.9)	38 (58.5)	36 (55.4)		
Smoking status, *n* (%)	No	53 (40.8)	28 (43.1)	25 (38.5)	0.488	X^2^
	Yes	29 (22.3)	13 (20.0)	16 (24.6)		
	Unknown	48 (36.9)	25 (38.5)	23 (35.4)		
BMI, *n* (%)	≥25	61 (46.9)	27 (41.5)	34 (52.3)	0.561	X^2^
	<25	44 (33.8)	22 (33.8)	22 (33.8)		
	Unknown	25 (19.2)	16 (24.6)	9 (13.8)		
Mucinous component, *n* (%)	No	80 (61.5)	42 (64.6)	38 (58.5)	0.835	X^2^
	Yes	22 (16.9)	11 (16.9)	11 (16.9)		
	Missing	28 (21.5)	12 (18.5)	16 (24.6)		
Tumor differentiation, *n* (%)	Well	56 (43.1)	29 (44.6)	27 (41.5)	0.726	X^2^
	Moderate or poor	25 (19.2)	14 (21.5)	11 (16.9)		
	Unknown	49 (37.7)	22 (33.8)	27 (41.5)		
Location of primary tumor, *n* (%)	Left colon	73 (56.2)	37 (56.9)	36 (55.4)	0.607	X^2^
	Right colon	35 (26.9)	19 (29.2)	16 (24.6)		
	Rectum	22 (16.9)	9 (13.8)	13 (20.0)		
Primary tumor resection **, *n* (%)	No	61 (46.9)	29 (44.6)	32 (49.2)	0.598	X^2^
	Yes	69 (53.1)	36 (55.4)	33 (50.8)		
No. of metastatic sites	1	91 (70.0)	44 (67.7)	47 (72.3)	0.566	X^2^
	≥2	39 (30.0)	21 (32.3)	18 (27.7)		
RAS status, *n* (%)	Mutant	60 (46.2)	30 (46.2)	30 (46.2)	0.862	X^2^
	Wild	64 (49.2)	31 (47.7)	33 (50.8)		
	Unknown	6 (4.6)	4 (6.2)	4 (6.2)		
BRAF status, *n* (%)	Mutant	3 (2.3)	1 (1.5)	2 (3.1)	0.618	Fisher’s Exact
	Wild	106 (81.5)	54 (83.1)	52 (80.0)		
	Unknown	21 (16.2)	10 (15.4)	11 (16.9)		
MSI status, *n* (%)	High	4 (3.1)	2 (3.1)	2 (3.1)	1.000	Fisher’s Exact
	Stable	106 (81.5)	54 (83.1)	42 (64.6)		
	Unknown	20 (15.4)	9 (13.8)	11 (16.9)		
CEA level, *n* (%)	<ULN	25 (19.2)	12 (18.5)	13 (20.0)	0.700	X^2^
	≥ULN	84 (64.6)	44 (67.7)	40 (61.5)		
	Unknown	21 (16.2)	9 (13.8)	12 (18.5)		
CA 19-9 level, *n* (%)	<ULN	45 (34.6)	24 (36.9)	21 (32.3)	0.672	X^2^
	≥ULN	67 (51.5)	33 (50.8)	34 (52.3)		
	Unknown	18 (13.8)	8 (12.3)	10 (15.4)		
Treatment regimens, *n* (%)	Oxaliplatin-based	88 (67.7)	45 (69.2)	43 (66.2)	0.708	X^2^
	Irinotecan-based	42 (32.3)	20 (30.8)	22 (33.8)		
Liver metastasis, *n* (%)	No	39 (30.0)	19 (29.2)	20 (30.8)	0.848	X^2^
	Yes	91 (70.0)	46 (70.8)	45 (69.2)		
Peritoneal metastasis, *n* (%)	No	94 (72.3)	46 (70.8)	48 (73.8)	0.695	X^2^
	Yes	36 (27.7)	19 (29.2)	17 (26.2)		

BMI: body mass index; CEA: Carcinoembryonic antigen; CA 19-9: Carbohydrate antigen 19-9; CAR: C-reactive protein (CRP)/albumin ratio; ECOG: Eastern Cooperative Oncology Group; MSI: Microsatellite instability; m: Mann–Whitney u; *n*: Number; ULN: Upper limit of normal; ** Primary tumor resection refers to surgery performed after systemic therapy in patients with de novo metastatic disease; X^2^: Chi-square.

**Table 3:** Univariate and multivariate Cox regression analyses for PFS in propensity-matched cohort.

	Univariate			Multivariate		
	HR	95%	*p*	HR	95%	*p*
Age (years) (<65 vs. ≥65)	0.937	0.642–1.368	0.737			
Gender (Female vs. Male)	0.908	0.625–1.320	0.613			
ECOG (0 vs. ≥1)	1.307	0.894–1.910	0.167			
Smoking status (No vs. Yes)	1.403	0.864–2.277	0.171			
BMI (≥25 vs. <25)	1.077	0.714–1.625	0.724			
Mucinous component (No vs. Yes)	1.450	0.932–2.255	0.099	2.335	1.178–4.628	***0.015***
Tumor differentiation (Well vs. Mod.-Poor)	1.139	0.776–1.671	0.507			
Primary tumor resection * (No vs. Yes)	1.229	0.848–1.781	0.277			
CAR Status (Low vs. High)	1.588	1.094–2.303	0.015			
RAS status (Wild vs. Mutant)	1.305	0.894–1.907	0.168			
BRAF status (Wild vs. Mutant)	1.955	0.617–6.193	0.254			
MSI status (High vs. Stable)	1.723	0.537–5.526	0.360			
CEA level (≤ULN vs. <ULN)	2.909	1.617–5.233	<0.001	2.231	1.017–4.895	***0.045***
CA 19-9 level (≤ULN vs. <ULN)	1.511	0.989–2.309	0.056			
Treatment regimens (Oxaliplatin vs. Irinotecan)	0.967	0.655–1.425	0.864			
No. of Metastatic Sites (1 vs. ≥2)	0.808	0.538–1.213	0.304			
Liver metastasis (No vs. Yes)	0.656	0.441–0.976	0.038			
Peritoneal metastasis (No vs. Yes)	0.857	0.560–1.312	0.477			

Bold-italic values indicate statistical significance (*p* < 0.05). BMI: body mass index; CA 19-9: carbohydrate antigen 19-9; CAR: C-reactive protein/albumin ratio; CEA: carcinoembryonic antigen; ECOG: Eastern Cooperative Oncology Group; HR: hazard ratio. * Primary tumor resection refers to surgery performed after systemic therapy in patients with de novo metastatic disease.

**Table 4:** Univariate and multivariate Cox regression analyses for OS in propensity-matched cohort.

	Univariate			Multivariate		
	HR	95%	*p*	HR	95%	*p*
Age (years) (<65 vs. ≥65)	1.063	0.721–1.567	0.757			
Gender (Female vs. Male)	0.983	0.669–1.446	0.932			
ECOG (0 vs. ≥1)	1.340	0.907–1.979	0.141			
Smoking status (No vs. Yes)	1.127	0.680–1.868	0.642			
BMI (≥25 vs. <25)	1.272	0.832- 1.947	0.267			
Mucinous component (No vs. Yes)	1.182	0.732–1.908	0.494			
Tumor differentiation (Well vs. Mod.-Poor)	1.078	0.727–1.597	0.710			
Primary tumor resection * (No vs. Yes)	1.296	0.882–1.905	0.187			
CAR Status (Low vs. High)	1.701	1.155–2.505	0.007	1.792	1.157–2.775	***0.009***
RAS status (Wild vs. Mutant)	1.408	0.951–2.083	0.087			
BRAF status (Wild vs. Mutant)	4.171	1.282–13.568	0.018	4.543	1.070–19.287	***0.040***
MSI status (High vs. Stable)	2.514	0.613–10.313	0.200			
CEA level (≤ULN vs. <ULN)	3.034	1.684–5.468	<0.001	3.331	1.830–6.065	***<0.001***
CA 19-9 level (≤ULN vs. <ULN)	1.645	1.062–2.549	0.026			
Treatment regimens (Oxaliplatin vs. Irinotecan)	1.017	0.683–1.516	0.932			
No. of Metastatic Sites (1 vs. ≥2)	0.905	0.595–1.377	0.641			
Liver metastasis (No vs. Yes)	0.932	0.617–1.409	0.738			
Peritoneal metastasis (No vs. Yes)	0.983	0.636–1.519	0.983			

Bold-italic values indicate statistical significance (*p* < 0.05). BMI: body mass index; CA 19-9: carbohydrate antigen; CAR: C-reactive protein/albumin ratio; CEA: carcinoembryonic antigen; ECOG: Eastern Cooperative Oncology Group; HR: hazard ratio. Primary tumor resection refers to surgery performed after systemic therapy in patients with de novo metastatic diseases. * Primary tumor resection refers to surgery performed after systemic therapy in patients with de novo metastatic disease.

## Data Availability

The datasets generated and analyzed during the current study are available from the corresponding author upon reasonable request.

## Supplementary Materials

The following supporting information can be downloaded at https://www.mdpi.com/article/10.3390/cancers18152425/s1, Table S1: Treatment interventions of low-CAR and high-CAR groups. Data S1: STROBE Statement—Checklist of items that should be included in reports of cohort studies.

## Abbreviations

AUC	Area Under the Curve
BMI	Body Mass Index
BRAF	B-Raf Proto-Oncogene, Serine/Threonine Kinase
CA 19-9	Carbohydrate Antigen 19-9
CAR	C-Reactive Protein-to-Albumin Ratio
CEA	Carcinoembryonic Antigen
CI	Confidence Interval
CRP	C-Reactive Protein
CRC	Colorectal Cancer
dMMR	Deficient Mismatch Repair
ECOG	Eastern Cooperative Oncology Group
EGFR	Epidermal Growth Factor Receptor
FCS	Fully Conditional Specification
HR	Hazard Ratio
ICI	Immune Checkpoint Inhibitor
IL	Interleukin
mCRC	de novo Metastatic Colorectal Cancer
MICE	Multivariate Imputation by Chained Equations
MSI	Microsatellite Instability
OS	Overall Survival
PFS	Progression-Free Survival
PS	Propensity Score
PSM	Propensity Score Matching
RAS	Rat Sarcoma Viral Oncogene
ROC	Receiver Operating Characteristic
SD	Standard Deviation
SMD	Standardized Mean Difference
SPSS	Statistical Package for the Social Sciences
TNF-α	Tumor Necrosis Factor Alpha
ULN	Upper Limit of Normal
VEGFR	Vascular Endothelial Growth Factor Receptor
