# Supplementary material for: Does the CRP/Albumin Ratio Independently Predict Survival in Metastatic Colorectal Cancer? Insights from a Propensity Score-Matched Cohort
Source: Cancers (Basel). 2026 Jul 28;18(15):2425. doi: 10.3390/cancers18152425 (PMC13465461; doi:10.3390/cancers18152425)
Supplement: Supplementary file 1 [file cancers-18-02425-s001.zip › Supplementary Data S1.pdf]

STROBE Statement—Checklist of items that should be included in reports of cohort studies

Manuscript: "Does the CRP/Albumin Ratio Independently Predict Survival in Metastatic Colorectal Cancer? Insights from a Propensity Score-Matched Cohort"

Item No	Recommendation	Location in manuscript
**Title and Abstract**		
**1**	**(a)** Indicate the study's design with a commonly used term in the title or the abstract<br>**(b)** Provide in the abstract an informative and balanced summary of what was done and what was found	Title; Abstract
**Introduction**		
**2**	Background/rationale — Explain the scientific background and rationale for the investigation being reported	Introduction (Section 1)
**3**	Objectives — State specific objectives, including any prespecified hypotheses	Introduction, final paragraph (Section 1)
**Methods**		
**4**	Study design — Present key elements of study design early in the paper	Section 2.1
**5**	Setting — Describe the setting, locations, and relevant dates, including periods of recruitment, exposure, follow-up, and data collection	Section 2.1 (Jan 2013-May 2025); follow-up in Section 3.3
**6**	**(a)** Give the eligibility criteria, and the sources and methods of selection of participants; describe methods of follow-up<br>**(b)** For matched studies, give matching criteria and number of exposed and unexposed	Section 2.1 (eligibility); Section 2.4 & 3.1 (matching: 16 covariates, 1:1 nearest-neighbor, caliper; 65 pairs)
**7**	Variables — Clearly define all outcomes, exposures, predictors, potential confounders, and effect modifiers; give diagnostic criteria, if applicable	Section 2.1 (CAR, cutoff); Section 2.2 (PFS, OS)
**8***	Data sources/measurement — For each variable of interest, give sources of data and details of methods of assessment (measurement); describe comparability of assessment methods if there is more than one group	Section 2.1
**9**	Bias — Describe any efforts to address potential sources of bias	Section 2.4 (propensity score matching to reduce confounding)
**10**	Study size — Explain how the study size was arrived at	Section 2.1 / 3.1 (all consecutive eligible patients)
**11**	Quantitative variables — Explain how quantitative variables were handled in the analyses; if applicable, describe which groupings were chosen and why	Section 2.1 (CAR dichotomized at ROC/Youden-derived cutoff 2.69); Section 2.4, and as a continuous variable (Section 3.4)
**12**	**(a)** Describe all statistical methods, including those used to control for confounding<br>**(b)** Describe any methods used to examine subgroups and interactions<br>**(c)** Explain how missing data were addressed<br>**(d)** If applicable, explain how loss to follow-up was addressed<br>**(e)** Describe any sensitivity analyses	Section 2.4 — (a) logistic PS model, KM/log-rank, multivariate Cox; (c) multiple imputation (FCS/chained equations, 5 datasets; imputed versions of residually imbalanced covariates); (d) censoring in Section 2.2; (b),(e) section 3.4. (Continuous CAR sensitivity analysis)
**Results**		
**13***	**(a)** Report numbers of individuals at each stage of study—e.g., numbers potentially eligible, examined for eligibility, confirmed eligible, included in the study, completing follow-up, and analysed<br>**(b)** Give reasons for non-participation at each stage<br>**(c)** Consider use of a flow diagram	Section 3.1 (n=212 included; 65 matched pairs / 130 analysed); exclusion criteria in Section 2.1

Item No	Recommendation	Location in manuscript
**14***	**(a)** Give characteristics of study participants and information on exposures and potential confounders<br>**(b)** Indicate number of participants with missing data for each variable of interest<br>**(c)** Summarise follow-up time	Table 1, Table 2, Section 3.1; missing-data ("Unknown/Missing") categories shown in Tables 1–2; follow-up in Section 3.3
**15***	Outcome data — Report numbers of outcome events or summary measures over time	Section 3.3 (88.2% progression; median PFS/OS)
**16**	**(a)** Give unadjusted estimates and, if applicable, confounder-adjusted estimates and their precision (e.g., 95% CI); make clear which confounders were adjusted for and why they were included<br>**(b)** Report category boundaries when continuous variables were categorized<br>**(c)** If relevant, consider translating estimates of relative risk into absolute risk for a meaningful time period	Tables 3 & 4 (univariate + multivariate Cox, HR with 95% CI); category boundary (CAR 2.69) in Section 2.1; median PFS/OS in Section 3.3
**17**	Other analyses — Report other analyses done—e.g., analyses of subgroups and interactions, and sensitivity analyses	Section 3.3 (comparison of unmatched vs. propensity-matched cohorts), section 3.4. (Continuous CAR sensitivity analysis)
**Discussion**		
**18**	Key results — Summarise key results with reference to study objectives	Discussion (Section 4), first paragraph
**19**	Limitations — Discuss limitations of the study, taking into account sources of potential bias or imprecision; discuss both direction and magnitude of any potential bias	Discussion, limitations paragraph (Section 4)
**20**	Interpretation — Give a cautious overall interpretation of results considering objectives, limitations, multiplicity of analyses, results from similar studies, and other relevant evidence	Discussion (Section 4), Conclusion (Section 5)
**21**	Generalisability — Discuss the generalisability (external validity) of the study results	Discussion (Section 4), paragraph on inclusion of both resectable and unresectable disease
**Other Information**		
**22**	Funding — Give the source of funding and the role of the funders for the present study and, if applicable, for the original study on which the present article is based	Funding statement

**Give information separately for exposed and unexposed groups.*

Note: An Explanation and Elaboration article discusses each checklist item and gives methodological background and published examples of transparent reporting. The STROBE checklist is best used in conjunction with this article (freely available at https://www.strobe-statement.org).
