# Supplementary material for: Does the CRP/Albumin Ratio Independently Predict Survival in Metastatic Colorectal Cancer? Insights from a Propensity Score-Matched Cohort
Source: Cancers (Basel). 2026 Jul 28;18(15):2425. doi: 10.3390/cancers18152425 (PMC13465461; doi:10.3390/cancers18152425)
Supplement: Supplementary file 1 [file cancers-18-02425-s001.zip › Supplementary Table S1.pdf]

Table S1. Treatment interventions of low-CAR and high-CAR groups.

Treatment interventions	Regimen	Low-CAR (n=88) n, %	High-CAR (n=124) n, %	*p*-value
First-line chemotherapy	FOLFOX	43 (48.9)	54 (43.5)	0.284
	FOLFIRI	23 (26.1)	45 (36.3)	
	CAPOX	22 (25)	25 (20.2)	
First-line biologic agents	Bevacizumab	67 (76.1)	88 (71)	0.101
	Cetuximab	19 (21.6)	24 (19.3)	
	Panitumumab	2 (2.3)	12 (9.7)	
Second-line	FOLFOX	10 (15.6)	15 (16.5)	0.937
	FOLFIRI	52 (81.3)	73 (80.2)	
	CAPOX	2 (3.1)	2 (2.2)	
	Capecitabine	0	1 (1.1)	
Third line	CT rechallenge	13 (41.9)	18 (58.1)	0.630
	Regorafenib	20 (47.6)	22 (52.4)	

CAR: C-reactive protein albumin ratio; CT: chemotherapy; FOLFIRI: Folinic acid, 5-FU, and irinotecan; FOLFOX: Folinic acid, 5-FU, and oxaliplatin CAPOX: Capecitabine and oxaliplatin.
